# Comparative evaluation of commercially available point-of-care heartworm antigen tests using well-characterized canine plasma samples

**DOI:** 10.1186/s13071-017-2447-3

**Published:** 2017-11-09

**Authors:** Lindsay A. Starkey, Joy V. Bowles, Mark E. Payton, Byron L. Blagburn

**Affiliations:** 1 Department of Pathobiology, College of Veterinary Medicine, Auburn University, Auburn, AL 36849 USA; 2 Department of Statistics, Oklahoma State University, Stillwater, OK USA

**Keywords:** Antigen, Canine, *Dirofilaria immitis*, Heartworm, Heat treatment

## Abstract

**Background:**

*Dirofilaria immitis* is a worldwide parasite that is endemic in many parts of the United States. There are many commercial assays available for the detection of *D. immitis* antigen, one of which was modified and has reentered the market. Our objective was to compare the recently reintroduced Witness® Heartworm (HW) Antigen test Kit (Zoetis, Florham Park, NJ) and the SNAP® Heartworm RT (IDEXX Laboratories, Inc., Westbrook, ME) to the well-based ELISA DiroChek® Heartworm Antigen Test Kit (Zoetis, Florham Park, NJ).

**Methods:**

Canine plasma samples were either received at the Auburn Diagnostic Parasitology Laboratory from veterinarians submitting samples for additional heartworm testing (*n* = 100) from 2008 to 2016 or purchased from purpose-bred beagles (*n* = 50, presumed negative) in 2016. Samples were categorized as “positive,” “borderline” or “negative” using our established spectrophotometric cutoff value with the DiroChek® assay when a sample was initially received and processed. Three commercially available heartworm antigen tests (DiroChek®, Witness® HW, and SNAP® RT) were utilized for simultaneous testing of the 150 samples in random order as per their package insert with the addition of spectrophotometric optical density (OD) readings of the DiroChek® assay. Any samples yielding discordant test results between assays were further evaluated by heat treatment of plasma and retesting. Chi-square tests for the equality of proportions were utilized for statistical analyses.

**Results:**

Concordant results occurred in 140/150 (93.3%) samples. Discrepant results occurred in 10/150 samples tested (6.6%): 9/10 occurring in the borderline heartworm (HW) category and 1/10 occurring in the negative HW category.

The sensitivity and specificity of each test compared to the DiroChek® read by spectrophotometer was similar to what has been reported previously (Witness®: sensitivity 97.0% [94.1–99.4%], specificity 96.4% [95.5–100.0%]; SNAP® RT: sensitivity 90.9% [78.0–100.0%], specificity 98.8% [96.0–100.0%]). There were significant differences detected when comparing the sensitivities of the SNAP® RT and the Witness® HW to the DiroChek® among the 150 total samples (*p* = 0.003) and the 50 “borderline” samples (*p* = 0.001).

**Conclusions:**

In this study, the sensitivity of the Witness® HW was higher than the sensitivity of the SNAP® RT when compared with the DiroChek® test results prior to heat treatment of samples.

## Background

*Dirofilaria immitis*, the causative agent of heartworm, was first described in the United States, but it has expanded its distribution and is now considered a global, potentially life-threatening parasite of dogs and cats [1–4]. Diagnosis of *D. immitis* infection is multifaceted; however, the mainstay of current heartworm testing relies upon detection of circulating antigen present in a whole blood, plasma, or serum sample [1, 5]. A number of point-of-care and reference laboratory diagnostic assays are available for the detection of *D*. *immitis* antigen, all with varying reported sensitivities and specificities that can be decreased when testing animals infected with few adult worms or infected with certain other parasites, respectively [6–10]. Additionally, presence of immune-complexes can complicate the diagnosis of heartworm infection when relying on an antigen-based diagnostic assay; several recent publications have reported an increase in the number of antigen-positive samples in both dogs and cats following pretreatment of the serum or plasma sample with heat [11–14].

The objective of this study was to evaluate the current Witness® Heartworm (HW) Antigen Test Kit (Zoetis, Florham Park, NJ). This point-of-care assay was initially launched in the United States in 1997, modified in 2013 to improve test performance and sensitivity, and most recently re-released in early 2016 after technical improvements to increase test specificity and reduce false-positive test results [10]. To best evaluate the performance of the Witness® HW assay, we chose to test canine plasma samples in order to compare the results between the Witness® HW, another point-of-care antigen test (SNAP® Heartworm RT, IDEXX Laboratories, Inc., Westbrook, ME), and a well-based ELISA heartworm antigen detection assay (DiroChek® Heartworm Antigen Test Kit, Zoetis, Florham Park, NJ).

## Methods

### Samples, initial heartworm testing, and sample categorization

From 2008 through 2016, veterinarians submitted canine plasma samples to the Auburn University Diagnostic Parasitology Laboratory service to be tested for the presence of *D. immitis* antigen using the DiroChek® Heartworm Antigen Test Kit.

Initial testing with the DiroChek® was performed according to manufacturer’s instructions with the addition of a spectrophotometric reading for determination of optical density. In short, 5 min following the addition of the final solution, the plate was examined for a clear to blue color change prior to placement in a spectrophotometric plate-reader (Synergy HTX Multi-Mode Microplate Reader, BioTek Instruments, Inc., Winooski, VT). Following initial testing, remaining plasma was stored at −20 °C.

The samples with sufficient volume (>500 μL) were placed into two categories (positive [*n* = 75] and borderline [*n* = 56]) based upon the spectrophotometric optical density (OD) value obtained during initial antigen testing with the DiroChek®. Samples were categorized as HW positive if the OD reading was greater than or equal to that of the concurrently tested positive control and borderline if the OD reading was greater than that of the concurrently tested negative control but at or below the established “cutoff” value (three standard deviations = 0.009). Fifty HW-positive samples and 50 borderline HW samples were randomly selected for inclusion in the study. Plasma was purchased for use as HW negative samples from purpose-bred beagles (*n* = 50) with a history of no exposure to mosquitoes or heartworm-infected animals. Once the 150 samples were identified for testing, the testing order was randomly assigned; and the personnel testing the samples were blinded to the initial heartworm antigen status of the samples.

### Serologic testing

Plasma samples (*n* = 150) were tested using three commercially available heartworm antigen tests: DiroChek®, Witness® HW, and the SNAP® Heartworm RT. Tests were performed, and antigen-positive or no detectable antigen (NDA) status was evaluated for each sample on each assay according to the manufacturer’s instructions. In addition to a visual determination of antigen-positive or NDA by color change on the DiroChek® as indicated in the manufacturer’s instructions, a spectrophotometric OD reading was obtained for each sample tested. All samples were tested in triplicate with the DiroChek® and in singlicate on the Witness® HW and the SNAP® RT. A designated person was assigned for each testing platform, and samples were tested and results were evaluated by that designated person for that testing platform for all 150 samples. Each sample was tested on all three assays simultaneously, and determination of each test result was completed individually by the dedicated person independent from the corresponding results on two other testing platforms.

### Heat treatment of samples

Following completion of testing for all 150 samples, any sample with discordant test results was subjected to heat treatment for dissociation of immune complexes as previously described [11, 13]. Briefly, each plasma sample was heated in a dry heat block at 104 °C for 10 min then centrifuged at 16,000 × *g*. The resulting supernatant was tested in singlicate on the three testing platforms (volume permitting) by the same testing and evaluation methods as described previously.

### Statistical analysis

Chi-square tests for the equality of proportions were utilized for statistical analyses with statistical significance at *P* < 0.05.

## Results

### Serologic testing

Testing of the 150 total samples yielded concordant results for 140/150 (93.3%) samples. Discordant testing results are presented in the table. All samples in the HW-positive category (50/50, 100.0%) tested positive on every testing platform. In the HW-negative category, 49/50 (98.0%) samples tested NDA on every testing platform; one sample tested NDA on the SNAP® RT and the DiroChek® while testing positive on the Witness® HW (see Table 1, sample 92). In the borderline HW group, 41/50 (82.0%) samples had concordant test results while 9/50 (18.0%; see table) had discordant results between the three testing platforms. Ten samples in the borderline HW group tested positive on all three platforms, and 31 samples tested NDA on all three platforms. Of the nine samples with discordant results, 8/9 (88.9%) tested NDA on the SNAP® RT while testing positive on one or both of the other testing platforms (samples 1, 23, 26, 52, 62, 68, 98 and 121). The other discordant borderline HW sample (1/9, 11.1%; sample 116) tested positive on the SNAP® RT while testing NDA on the other two testing platforms.

**Table 1:** Samples with discordant testing results prior to heat treatment (and results following heat treatment) for the three testing platforms

Sample^a^	HW Category^b^	Witness® HW	SNAP® RT	DiroChek®	Sample OD (+ control OD) Initial Test	Sample OD (+ control OD) Post-Heat Treatment
1	BL	+ (+)	− (vol.^b^)	+ (+)	0.038 (0.352)	0.049 (0.309)
23	BL	+ (+)	− (+)	+ (+)	0.013 (0.363)	0.070 (0.309)
26	BL	− (−)	− (−)	+ (−)	0.015 (0.363)	0.000 (0.309)
52	BL	+ (+)	− (+)	+ (+)	0.027 (0.470)	0.124 (0.309)
62	BL	+ (+)	− (+)	+ (+)	0.020 (0.470)	0.077 (0.309)
68	BL	− (+)	− (+)	+ (+)	0.019 (0.380)	0.050 (0.309)
92	Neg.	+ (−)	− (−)	− (−)	0.000 (0.380)	0.000 (0.309)
98	BL	+ (+)	− (+)	− (+)	0.004 (0.380)	0.025 (0.309)
116	BL	− (−)	+ (−)	− (−)	0.001 (0.337)	0.000 (0.309)
121	BL	+ (+)	− (vol.^c^)	− (+)	0.009 (0.337)	0.126 (0.309)

Sample OD values (DiroChek®) prior to and following heat treatment are included

^a^Sample number indicates the order in which samples were tested

^b^ *BL* borderline

^c^Insufficient volume (<150 μL) precluded testing on all testing platforms

### Heat treatment of samples

Ten samples (10/150; 6.6%) had discordant test results between the three testing platforms. Test results following heat treatment of the samples are presented in Table 1. Following heat treatment, 8/10 (80%) samples converted to concordant results on all three testing platforms. Due to volume restrictions (<150 μL), 2/10 samples were only tested following heat treatment on the DiroChek® and Witness® HW testing platforms, and concordance of the results on those two testing platforms was achieved.

### Statistical analysis

Among the 150 samples total and the 50 borderline samples, there were significant differences between the sensitivities of the SNAP® RT and the Witness® HW when compared with the DiroChek® (*p* = 0.003 and *p* = 0.001, respectively). No significant difference was present between the specificities of the SNAP® RT and the Witness® HW when compared to the DiroChek® (*p* = 0.244 and *p* = 0.238, respectively).

## Discussion

Overall, the majority (>90%) of the test results were in agreement with one another between the three assays. Test performance of the Witness® HW and the SNAP® RT were consistent with the reported sensitivity and specificity ranges of each assay: Witness® HW (sensitivity 97.0% [reported: 94.1–99.4%]; specificity 96.4% [reported: 95.5–100%]) and SNAP® RT (sensitivity 90.9% [reported: 78.0–100.0%]; specificity 98.8% [reported: 96.0–100.0%]) [9, 10, 15].

All of the samples categorized as positive HW samples tested positive on all three testing platforms; all but one of the samples categorized as negative HW samples tested NDA on all three assays, and 41/50 borderline HW samples had concordant results across all testing platforms.

Ten samples had results that were not consistent across the three testing platforms (Table 1). The single sample in the negative HW category with discordant results tested positive on the Witness® HW and NDA on both the SNAP® RT and the DiroChek® (sample 92). Heat treatment and retesting of that sample yielded NDA results on all three testing platforms. The postheating results taken together with the history of that dog (purpose-bred Class A beagle housed strictly indoors) would indicate that there was a false-positive result on the Witness® HW assay with this sample.

Of the nine discordant borderline HW samples, eight tested positive on one or two assays while testing NDA on the remaining assay(s) (samples 1, 23, 26, 52, 62, 68, 98 and 121); the NDA results on the assay(s) for these eight samples were presumed to be false-negative results based on the OD reading in conjunction with a positive visual result on at least one of the three testing platforms. Heat treatment of the eight samples with pursuant follow-up antigen testing was performed on all three assays for six samples (samples 23, 26, 52, 62, 68 and 98) and only on the Witness® HW and DiroChek® due to an insufficient amount of sample remaining following heat treatment on the two remaining samples (samples 1 and 121). The approximate volume of sample needed to run on the Witness® HW and the DiroChek® is one drop (~50 μL) each, while the SNAP® RT requires three drops totaling approximately 150 μL.

In all eight samples, testing of the heat-treated sample resulted in concordant test results between the assays (Table 1). Six of the eight samples were confirmed to be positive by conversion of an initial NDA result on an assay to a positive result after the sample was heat-treated (samples 23, 52, 62, 68, 98 and 121). One of the eight samples with insufficient postheating volume (sample 1) remained positive when tested postheating on the same assays with original positive results; insufficient volume precluded postheating testing on the SNAP® RT. The last of the eight samples (sample 26) tested NDA on all testing platforms following heat treatment. This sample was originally presumed to be testing false negative on the Witness® HW and SNAP® RT, as it tested positive on the DiroChek® and had a positive OD value; test results following heat treatment suggest a probable false-positive test result on the initial DiroChek® reading for that sample.

The last discordant borderline HW sample was presumed to be testing false positive on the SNAP® RT as the initial results of the other two assays were NDA and the OD reading was almost equal to that of the negative control (sample 116). Following heat treatment, NDA results were obtained on all three testing platforms suggesting that the initial SNAP® RT test result was a false positive.

False-positive test results do occur rarely with these commercially available heartworm antigen assays as no assay is 100% specific in all situations [9, 10, 15]. There is evidence in the literature that infection with other parasites, such as *Spirocerca lupi* and *Angiostrongylus vasorum,* may induce a false-positive heartworm antigen test result on several of the commercially available test platforms as well [7, 8].

False-negative test results are more common than false positives as there are several situations that may cause an animal to test NDA while actually harboring *D. immitis* worms: low worm numbers, male-only infection, presence of immature worms, inappropriate handling or use of an assay, and/or presence of immune complexes.

In the past couple of years, there have been several publications in the peer-reviewed literature discussing the use of heat treatment to enhance the detection of antigen present in certain samples; however, routine heating of samples is not recommended by the American Heartworm Society as that testing strategy is not included in the label instructions for the assays, and it could interfere with detection of other agents when tested on the combination tests [5, 11–14]. Heat treatment of samples should only be considered in animals for which there is a strong clinical suspicion of heartworm infection (eg, history of absent or noncompliant use of preventives, radiographic or echocardiographic changes consistent with heartworm infection, or presence of microfilaria) [14].

## Conclusions

All three of the commercially available heartworm antigen detection assays utilized in this study were easy to use and performed well for the majority of the samples, with consistent results obtained between the three assays. Each assay appeared to have at least one false-positive and one false-negative result. Statistically significant differences were detected when comparing the sensitivities of the SNAP® RT and the Witness® HW to the Dirochek® among the 150 total samples (*p* = 0.003) and specifically with the 50 “borderline” samples (*p* = 0.001). In this study, the sensitivity of the Witness® HW was higher than the sensitivity of the SNAP® RT when compared with the DiroChek® test results prior to heat treatment of samples. Additionally, use of heat treatment in the samples with discordant results appeared to increase the sensitivity of detection of antigen on the assay(s) that initially had an NDA test result. The OD values in antigen-positive samples also increased following heat treatment of the samples.

## Abbreviations

BL: Borderline
HW: Heartworm
NDA: No detectable antigen
OD: Optical density
